# Urinary NT-proBNP as a noninvasive biomarker for pediatric septic shock: a comparative study with serum levels

**DOI:** 10.1186/s12887-026-07371-8

**Published:** 2026-07-29

**Authors:** Fauzi, Faisal, Rahmat Budi Kuswiyanto, Rini Rossanti, Dzulfikar Djalil Lukmanul Hakim, Sri Endah Rahayuningsih

**Affiliations:** Department of Child Health, Faculty of Medicine, Universitas Padjadjaran/Dr. Hasan Sadikin General Hospital, Bandung, West Java Indonesia

**Keywords:** NT-proBNP, Pediatric sepsis, Septic shock, Urinary biomarker, Phoenix sepsis score

## Abstract

**Background:**

The 2024 Phoenix Sepsis Score (PSS) may not fully capture subclinical cardiovascular stress in pediatric sepsis. N-terminal pro–B-type natriuretic peptide (NT-proBNP), released under haemodynamic strain, is measurable in urine and serum and has been associated with cardiovascular dysfunction. This study aimed to compare urinary and serum NT-proBNP levels in pediatric sepsis and septic shock, evaluate their ability to differentiate these conditions, and examine their correlations with PSS.

**Methods:**

Participants aged 1 month–18 years with sepsis or septic shock according to PSS were included in this prospective cross-sectional analytical study at a tertiary hospital in Bandung, Indonesia. Urinary and serum NT-proBNP concentrations were measured within 48 h of diagnosis. Patients with preexisting heart, kidney, or urological problems were excluded. Main analyses included Mann-Whitney U, ROC curves, and Spearman correlations. To evaluate statistical stability, 1,000 bias-corrected and accelerated (BCa) bootstrap iterations, fragility index (FI) analysis, and time-adjusted sensitivity analyses were added.

**Results:**

Final analyses comprised 52 children (26 sepsis, 26 septic shock). Median urinary NT-proBNP levels were higher in septic shock than in sepsis (8,065 vs. 210 pg/mL; *p* = 0.003), as were serum NT-proBNP levels (1,655 vs. 200 pg/mL; *p* = 0.005). Urinary NT-proBNP had an AUC of 0.743 (95% BCa CI 0.584–0.867) for septic shock (cut-off > 9,550 pg/mL, specificity 100%, sensitivity 42.3%, FI = 4), whereas serum NT-proBNP showed an AUC of 0.726 (95% BCa CI 0.573–0.852; cut-off > 1,640 pg/mL, specificity 92.3%, sensitivity 50.0%, FI = 3). Sensitivity analyses confirmed performance stability when restricting collection to ≤ 6 h. Urinary NT-proBNP correlated weakly with PSS (*r* = 0.261; *p* = 0.031), while serum NT-proBNP did not (*r* = 0.221; *p* = 0.058).

**Conclusions:**

Urinary NT-proBNP demonstrated moderate discriminatory ability and high specificity for pediatric septic shock, comparable to serum NT-proBNP, although its correlation with PSS was modest. It may have potential value as an adjunctive rule-in biomarker for cardiovascular stress in pediatric sepsis, but not as a screening tool or replacement for clinical assessment. Further studies are needed to determine its broader clinical utility in pediatric sepsis.

**Supplementary Information:**

The online version contains supplementary material available at https://doi.org/10.1186/s12887-026-07371-8.

## Background

Sepsis continues to contribute considerably to pediatric morbidity and mortality worldwide [1]. Globally, there are approximately 20.3 million cases of sepsis in children under five years of age and 4.9 million cases in those aged 5–19 years annually [2]. The mortality rate is estimated to range from 10% to 50%, with the greatest impact in low- to middle-income countries due to limited healthcare facilities. In Indonesia, pediatric sepsis mortality has been reported to range from 37.3% to 65%, highlighting the need for improved early classification of sepsis severity [3–5]. 

Septic shock represents the most advanced stage of the spectrum marked by profound circulatory disturbance. Preceding that is a condition called septic cardiomyopathy, a reversible heart dysfunction of weakened heart muscle contractility and/or stiffness, often with ventricular dilation, and it can appear early in sepsis, leading to cardiovascular failure and poorer outcomes. The Phoenix Sepsis Score (PSS) has been validated internationally since 2024 for pediatric sepsis classification. Sepsis is defined as suspected or confirmed infection and a PSS score of ≥ 2 points, whereas septic shock is sepsis that includes ≥ 1 point in the cardiovascular domain. Although PSS improves previous sepsis criteria, it focuses on global organ dysfunction and may not fully capture isolated myocardial stress in children with sepsis [6]. Consequently, adjunctive biomarkers for identifying cardiovascular involvement in pediatric sepsis, particularly noninvasive ones, are needed.

Alongside biomarker-based approaches, interest is also growing in artificial intelligence (AI)-assisted tools for pediatric risk stratification. Recent studies have shown that machine learning models and large language models can achieve diagnostic performance comparable to experienced pediatricians in identifying serious bacterial infections, suggesting a potential role as adjunctive clinical decision-support systems [7]. In parallel, noninvasive biomarker research has expanded to alternative biological specimens such as saliva and urine. Salivary biomarkers, including CRP and other inflammatory mediators, have shown promise in pediatric infectious and inflammatory conditions, reflecting a broader movement toward child-friendly diagnostic approaches [8]. Together, these developments highlight ongoing efforts to improve early recognition and risk assessment in pediatric sepsis through complementary advances in computational and biomarker-based methods.

N-terminal pro–B-type natriuretic peptide (NT-proBNP) is a peptide released by cardiomyocytes in response to ventricular wall stretch and increased haemodynamic stress associated with systemic inflammation and impaired tissue perfusion during sepsis. Elevated serum NT-proBNP levels are consistently higher in both adults and children with septic shock [9, 10]. After secretion, NT-proBNP from the bloodstream then enters the kidneys and is partially excreted into the urine. Urine testing is noninvasive and advantageous in resource-limited settings as well as in children with difficult vascular access [11, 12]. However, research on urinary NT-proBNP in children with sepsis is still limited.

Urinary NT-proBNP is not intended to replace established clinical assessment or urgent vascular-based management in septic shock. Given that vascular access is commonly established early during shock resuscitation, particularly in critically ill children, urinary NT-proBNP may be more appropriately positioned as an adjunctive cross-sectional discrimination rather than a method for acute shock identification. Its potential clinical role may lie in providing additional information regarding cardiovascular stress alongside existing clinical evaluation, particularly in settings where noninvasive assessment is advantageous or where blood sampling is undesirable. Given the need for improved clinical assessment of sepsis and the practical use of urine sampling, this study focused on evaluating urinary and serum NT-proBNP as biomarkers for pediatric septic shock and analysing their correlation with sepsis severity as measured by PSS.

## Materials and methods

### Study aim

We aimed to compare urinary and serum NT-proBNP levels between pediatric sepsis and septic shock, evaluate their performance in discriminating the two conditions, and analyse their correlations with sepsis severity as measured by PSS. The intended clinical application was adjunctive classification of septic shock, and not replacement of standard clinical assessment, acute shock identification, or treatment decision making. Urinary NT-proBNP was evaluated as a complementary noninvasive biomarker that may provide additional information regarding cardiovascular stress alongside established clinical parameters.

### Study design

From December 2025 to January 2026, at Bandung, Indonesia’s tertiary referral centre Dr. Hasan Sadikin General Hospital, we conducted a prospective cross-sectional analytical study to evaluate urinary and serum NT-proBNP concentrations in discriminating pediatric septic shock from sepsis. We also analysed their correlations with PSS. The inclusion criteria were for children hospitalized with sepsis or septic shock, with sample measurements collected within 48 h of diagnosis. Sepsis was defined as suspected or confirmed infection with a PSS of 2 points or higher, and septic shock was defined as sepsis that includes a cardiovascular domain score of 1 point or higher. The ages of the children varied from one month old to eighteen years old. All the individuals’ legal guardians gave their written informed consent. The exclusion criteria were patients who had previously been diagnosed with severe heart failure, pulmonary hypertension, dilated cardiomyopathy, or postcardiac surgery in less than 1 month; who had chronic kidney disease (CKD) stages III-V; who had severe or stage III acute kidney injury (AKI); who had undergone renal replacement therapy (RRT or dialysis); and who had a history of urological diseases such as urinary tract obstruction, hydronephrosis, or anatomical abnormalities of the urinary tract.

### Sample size determination

Sample size estimation was calculated to evaluate differences in NT-proBNP levels between pediatric sepsis and septic shock patients. The calculation was based on a comparison of two independent numerical means via an unpaired difference test. Based on previous evidence showing that NT-proBNP concentrations tend to be higher in patients with greater cardiovascular dysfunction and greater illness severity in sepsis, we prespecified a directional hypothesis and used a one-sided significance threshold for sample size estimation. Nevertheless, we acknowledge that one-sided assumptions in clinical biomarker research may produce more optimistic sample size estimates. A 5% one-sided significance threshold was used (α = 0.05; Zα = 1.64), along with an 80% statistical power (β = 0.20; Zβ = 0.84). As no prior pediatric studies were available to provide reliable estimates of expected NT-proBNP differences between pediatric sepsis and septic shock groups, an effect size approach was applied. A standardized effect size of 0.7 was selected to represent a moderate-to-large difference considered clinically meaningful for exploratory biomarker evaluation, while acknowledging the limited availability of comparable reference data. With these assumptions, the minimum required sample size was 26 subjects per group. Thus, a total of 52 children were required, equally distributed between the sepsis and septic shock groups.

The sample size for the correlation between NT-proBNP concentrations and PSS was based on an assumed moderate correlation effect size of 0.4, as no prior studies have been conducted. With a two-sided significance level of 5% (α = 0.05; Zα = 1.96) and a statistical power of 80% (β = 0.20; Zβ = 0.84), the sample size needed was a minimum of 47 subjects. Considering that a total of 52 children were assessed for the difference test, the sample size needed was sufficient for another correlation test.

### Data collection

Participants were recruited via consecutive sampling method. Children admitted to the pediatric emergency ward, pediatric general wards, pediatric high care unit (PHCU), or pediatric intensive care unit (PICU) at Dr. Hasan Sadikin General Hospital, Bandung, during the study period with suspected or confirmed infection who subsequently developed organ dysfunction fulfilling Phoenix pediatric sepsis criteria were eligible for data collection and study analyses. The major study variables were NT-proBNP levels in urine and serum, which were analysed within 48 h after sepsis or septic shock diagnosis. Using a Chinese-made Elabscience^®^ human NT-proBNP research kit, the NT-proBNP levels were determined by enzyme-linked immunosorbent assay (ELISA). The same assay kit was used for both urinary and serum measurements. The assay reported an analytical sensitivity of 90 pg/mL and a validated range of 160–10,000 pg/mL. A total of 17 serum and 16 urinary measurements were below the validated range (< 160 pg/mL), whereas 2 serum and 10 urinary measurements exceeded the upper validated range (> 10,000 pg/mL). In accordance with the manufacturer’s protocol, numerical values generated by the assay were recorded as reported. No additional dilution or repeat measurement was performed for samples exceeding the validated assay range.

Both urinary and serum samples were processed immediately after collection. Specimens were centrifuged at 3,000 rpm for 15 min, and the supernatant was aliquoted and stored at − 80 °C until analysis. After all samples had been collected, aliquots were thawed at room temperature (approximately 25 °C) for approximately 2 h before assay and analysed in the same batch. No repeated freeze-thaw cycles were performed prior to analysis. Samples were analysed according to the assay manufacturer’s instructions, and measurable concentrations generated by the assay were recorded for statistical analyses.

Age, estimated filtration glomerular rate (eGFR), and sampling time were considered potential confounders. Clinical and demographic data, including sex, nutritional status, source of infection, site of initial care, use of respiratory support, vasopressor use, hemodynamic parameters, and microbiological culture results were also simultaneously obtained. Disease severity classification into sepsis and septic shock based on PSS criteria, as well as the total PSS score, were considered the primary outcomes. There were no changes to the study methods or outcomes after the study commenced. Only complete datasets were included in the final analyses. Since there was no missing data for the variables included in this study, we did not need to perform any data imputation.

### Participant selection

**Fig. 1:**
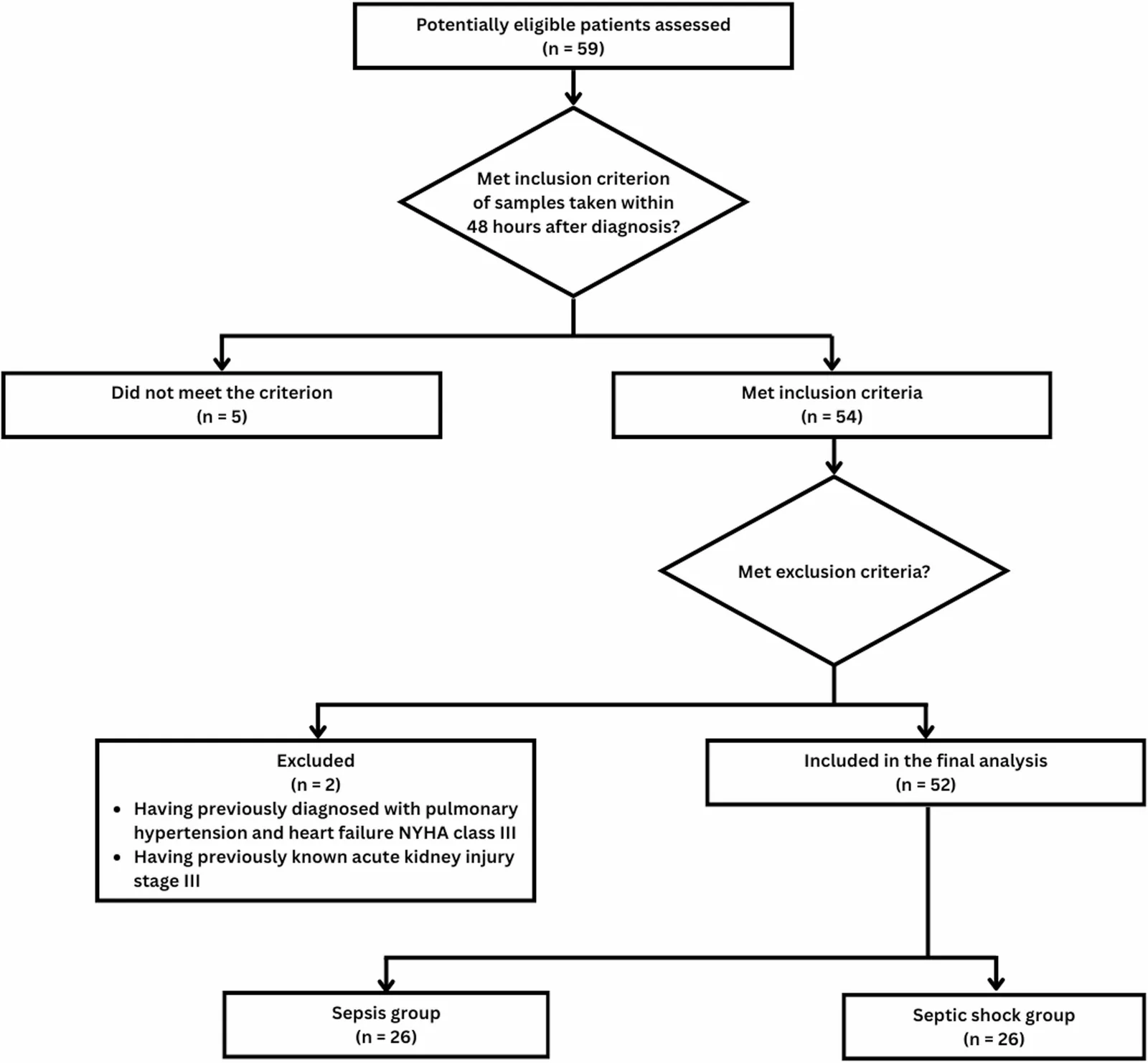
Flow diagram of participant selection

Fifty-nine potentially eligible patients were assessed in this study, but 5 did not meet the criterion of having both urine and serum samples taken within 48 h after sepsis diagnosis based on PSS. Among the 54 subjects who met the inclusion criteria, 2 were excluded based on predefined exclusion criteria: 1 due to pulmonary hypertension with New York Heart Association (NYHA) class III heart failure and 1 due to acute kidney injury stage III. Taken together, 52 patients were put in the final analyses and were evenly split into 26 sepsis and 26 septic shock groups. Figure 1 shows the participant selection flow diagram.

### Outcome of the study

The primary outcome of this study was pediatric septic shock status, defined according to PSS criteria. This status was used to evaluate the discriminative performance of urinary and serum NT-proBNP concentrations in distinguishing pediatric sepsis from septic shock. Discriminative ability was assessed using receiver operating characteristic (ROC) analysis and area under the curve (AUC) estimates.

The secondary outcomes included: (1) comparison of urinary and serum NT-proBNP concentrations between children with sepsis and septic shock, and (2) assessment of the correlations between NT-proBNP concentrations, in both urine and serum, and sepsis severity as measured by PSS. Septic shock classification according to the PSS criteria was also used as the grouping variable for comparative analyses.

### Statistical analyses

We used SPSS (Statistical Package for the Social Sciences) 27.0 to analyse these data sets. Descriptive statistics were used for subject characteristics, and the Shapiro-Wilk test was employed to ensure data normality. We used the Mann-Whitney U test, which is applicable to data that does not adhere to a normal distribution, to compare the quantities of NT-proBNP in the urine and serum between the sepsis and septic shock groups. To determine the discriminative ability of urinary and serum NT-proBNP levels to distinguish pediatric septic shock from sepsis, this study used receiver operating characteristic (ROC) analysis. Values of significance and area under the curve (AUC) with confidence intervals (CIs) were used to evaluate the discriminative performance results. The link between NT-proBNP levels and PSS was examined using Spearman rank correlation, which is useful when dealing with nonnormally distributed data. We considered a *p* value of less than 0.05 to be statistically significant. In view of the skewed NT-proBNP distributions, logarithmic scales were used for graphical representations where appropriate. To evaluate the robustness of ROC estimates given the relatively small sample size, bootstrap resampling with 1,000 bias-corrected and accelerated (BCa) iterations was performed. In addition, fragility analysis was conducted for statistically significant dichotomous associations using the Fragility Index (FI) and Fragility Quotient (FQ). Sensitivity analyses were also performed by adjusting for sampling time using logistic regression models and by restricting analyses to patients whose samples were collected within 6 h after sepsis diagnosis. Because urine and serum samples were not always collected simultaneously, the ≤ 6-hour sensitivity analyses were performed separately for each biomarker based on its respective sampling time. Consequently, the urinary and serum early-sampling subgroups were not necessarily composed of identical participants.

## Results

### Subject characteristics

A total of 52 children were enrolled in the final analyses. The study population was predominantly male (65.4%). In terms of potential confounders, there were no significant differences between the sepsis and septic shock groups regarding age (6.74 ± 5.58 vs. 6.86 ± 5.91 years, respectively; *p* = 0.941) or eGFR (113.23 ± 50.24 vs. 107.17 ± 53.48; *p* = 0.676). The median time from diagnosis to serum sampling was 1 h (range 0–48 h) in the sepsis group and 0 h (range 0–2 h) in the septic shock group (*p* = 0.109). The median time from diagnosis to urine sampling was 6 h (range 1–41 h) in the sepsis group and 4.5 h (range 1–44 h) in the septic shock group (*p* = 0.782). No statistically significant differences in sampling times were observed between groups. According to anthropometric measures and nutritional status, such as weight-for-age, length/height-for-age, and weight-for-length/weight-for-height/body mass index-for-age, malnutrition was common in both the sepsis and septic shock groups. Pneumonia was the frequent source of infection in 57.7% of patients with sepsis or septic shock, followed by urinary tract infections in 11.5%. Although patients’ initial treatment settings varied, 67.3% received their care in either the PHCU or the PICU. The septic shock group was more likely to utilize invasive mechanical ventilation (53.8% vs. 23.1% in the sepsis group). Vasopressor use was observed in 84.6% of the septic shock patients, of whom 57.7% received adrenaline. Moreover, impaired pupillary light reflexes were found in 34.6% of the septic shock group. Mean arterial pressure (MAP) was lower in the septic shock group than in the sepsis group (median 52.7 vs. 72.7 mmHg), accompanied by a higher total Phoenix Sepsis Score (median 5 vs. 2). In terms of microbiological characteristics, the overall culture positivity rate was 46.2%, most cultures were drawn from blood (73.1%), and multidrug-resistant organism (MDRO) status was observed in 30.8% of all cultures. Tables 1 and 2 present the detailed characteristics of the study population.

**Table 1:** Baseline characteristics

Variables	Total (*n* = 52)	Sepsis (*n* = 26)	Septic Shock (*n* = 26)
Sex, *n* (%)			
Male	34 (65.4)	19 (73.1)	15 (57.7)
Female	18 (34.6)	7 (26.9)	11 (42.3)
Nutritional Status (based on Weight-for-age), *n* (%)			
Normal	31 (59.6)	14 (53.8)	17 (65.4)
Underweight	7 (13.5)	4 (15.4)	3 (11.5)
Severely underweight	14 (26.9)	8 (30.8)	6 (23.1)
Nutritional Status (based on Length/height-for-age), *n* (%)			
Normal	26 (50.0)	10 (38.5)	16 (61.5)
Stunted	14 (26.9)	7 (26.9)	7 (26.9)
Severely Stunted	12 (23.1)	9 (34.6)	3 (11.5)
Nutritional Status (based on weight-for-length/height or BMI-for-age), *n* (%)			
Normal	28 (53.8)	15 (57.7)	13 (50.0)
Severely Wasted	12 (23.1)	5 (19.2)	7 (26.9)
Moderately Wasted	8 (15.4)	3 (11.5)	5 (19.2)
Risk of Overweight	1 (1.9)	0 (0.0)	1 (3.8)
Overweight	1 (1.9)	1 (3.8)	0 (0.0)
Obese	2 (3.8)	2 (7.7)	0 (0.0)
Initial diagnosis, *n* (%)			
Lower respiratory tract infection (pneumonia)	30 (57.7)	19 (73.1)	11 (42.2)
Urinary tract infection	6 (11.5)	2 (7.7)	4 (15.4)
Surgical site infection	4 (7.7)	1 (3.8)	3 (11.5)
Ventriculitis	2 (3.8)	0 (0.0)	2 (7.7)
Tracheostomy-associated infection	1 (1.9)	1 (3.8)	0 (0.0)
Other/unknown sources	9 (17.3)	3 (11.5)	6 (23.0)
Unit of care at initial diagnosis, *n* (%)			
Emergency ward	3 (5.8)	1 (3.8)	2 (7.7)
PHCU	19 (36.5)	13 (50.0)	6 (23.1)
PICU	16 (30.8)	6 (23.1)	10 (38.5)
General ward	14 (26.9)	6 (23.1)	8 (30.8)
Type of respiratory support at initial diagnosis, *n* (%)			
No invasive mechanical ventilation	32 (61.5)	20 (76.9)	12 (46.2)
Invasive mechanical ventilation	20 (38.5)	6 (23.1)	14 (53.8)
Use of vasopressor, *n* (%)			
Yes	22 (42.3)	0 (0.00)	22 (84.6)
No	30 (57.7)	0 (0.00)	4 (15.4)
Vasopressor drugs, *n* (%)			
Adrenaline	15 (28.8)	0 (0.00)	15 (57.7)
Adrenaline & Noradrenaline	4 (7.7)	0 (0.00)	4 (15.4)
Noradrenaline	3 (5.8)	0 (0.00)	3 (11.5)
No use of vasopressor	30 (57.7)	0 (0.00)	4 (15.4)
Pupillary light reflex, *n* (%)			
Reactive	42 (80.8)	25 (96.2)	17 (65.4)
Fixed pupils bilaterally	10 (19.2)	1 (3.8)	9 (34.6)
Culture results, *n* (%)			
Positive	24 (46.2)	11 (42.3)	13 (50.0)
Negative	22 (42.3)	12 (46.2)	10 (38.5)
Not performed	6 (11.5)	3 (11.5)	3 (11.5)
Culture specimens collected, *n* (%)			
Blood	37 (71.2)	17 (65.4)	20 (76.9)
Sputum	5 (9.6)	4 (15.4)	1 (3.8)
Urine	2 (3.8)	1 (3.8)	1 (3.8)
Cerebrospinal fluid	1 (1.9)	0 (0.0)	1 (3.8)
Sputum & blood	1 (1.9)	1 (3.8)	0 (0.0)
Not performed	6 (11.5)	3 (11.5)	3 (11.5)
Culture results (organism type), *n* (%)			
Negative culture or not performed	28 (53.8)	15 (57.7)	13 (50.0)
Gram-negative bacilli	11 (21.1)	4 (15.3)	7 (26.9)
Gram-positive cocci	7 (13.5)	4 (15.4)	3 (11.5)
Fungal	2 (3.8)	0 (0.0)	2 (7.7)
Polymicrobial	4 (7.6)	3 (11.5)	1 (3.8)
Culture result category, *n* (%)			
Negative culture or not performed	28 (53.8)	15 (57.7)	13 (50.0)
MDRO	16 (30.8)	10 (38.5)	6 (23.1)
Non-MDRO	6 (11.5)	1 (3.8)	5 (19.2)
Fungal	2 (3.8)	0 (0.0)	2 (7.7)
MAP (mmHg), Median (min–max)	70.0 (21.7–125.7)	72.7 (59.3–125.7)	52.7 (21.7–92.7)
Total Phoenix Sepsis Score, median (min–max)	3 (2–10)	2 (2–5)	5 (2–10)

*Abbreviations*: *n *frequency, *% *Percentage, *BMI *Body mass index, *PHCU *Pediatric high care unit, *PICU* Pediatric intensive care unit, *MDRO *Multidrug-resistant organism, *eGFR *Estimated glomerular filtration rate, *MAP *Mean arterial pressure

**Table 2:** Subject characteristics based on potential confounders

Variables	Total (*n* = 52)	Sepsis (*n* = 26)	Septic Shock (*n* = 26)	*P* value
Age (years), Mean ± SD	6.80 ± 5.69	6.74 ± 5.58	6.86 ± 5.91	0.941^a^
eGFR (mL/min/1.73 m^2^), Mean ± SD	110.20 ± 51.47	113.23 ± 50.24	107.17 ± 53.48	0.676^a^
Time from diagnosis to serum sampling (hours), median (min–max)	1 (0–48)	1 (0–48)	0 (0–2)	0.109^b^
Time from diagnosis to urine sampling (hours), median (min–max)	5 (1–44)	6 (1–41)	4.5 (1–44)	0.782^b^

*Abbreviations*: *n *frequency; *% *percentage, *eGFR *Estimated glomerular filtration rate

^a^Independent t-test

^b^Mann–Whitney U test; significant *p* ≤ 0.05

Regarding assay performance, 17 serum and 16 urinary NT-proBNP measurements were below the assay’s validated range (< 160 pg/mL), whereas 2 serum and 10 urinary measurements exceeded the upper validated range (> 10,000 pg/mL). All measurable values were retained for analysis without repeat testing after additional dilution.

### Differences in urinary and serum NT-proBNP levels between sepsis and septic shock in children

**Fig. 2:**
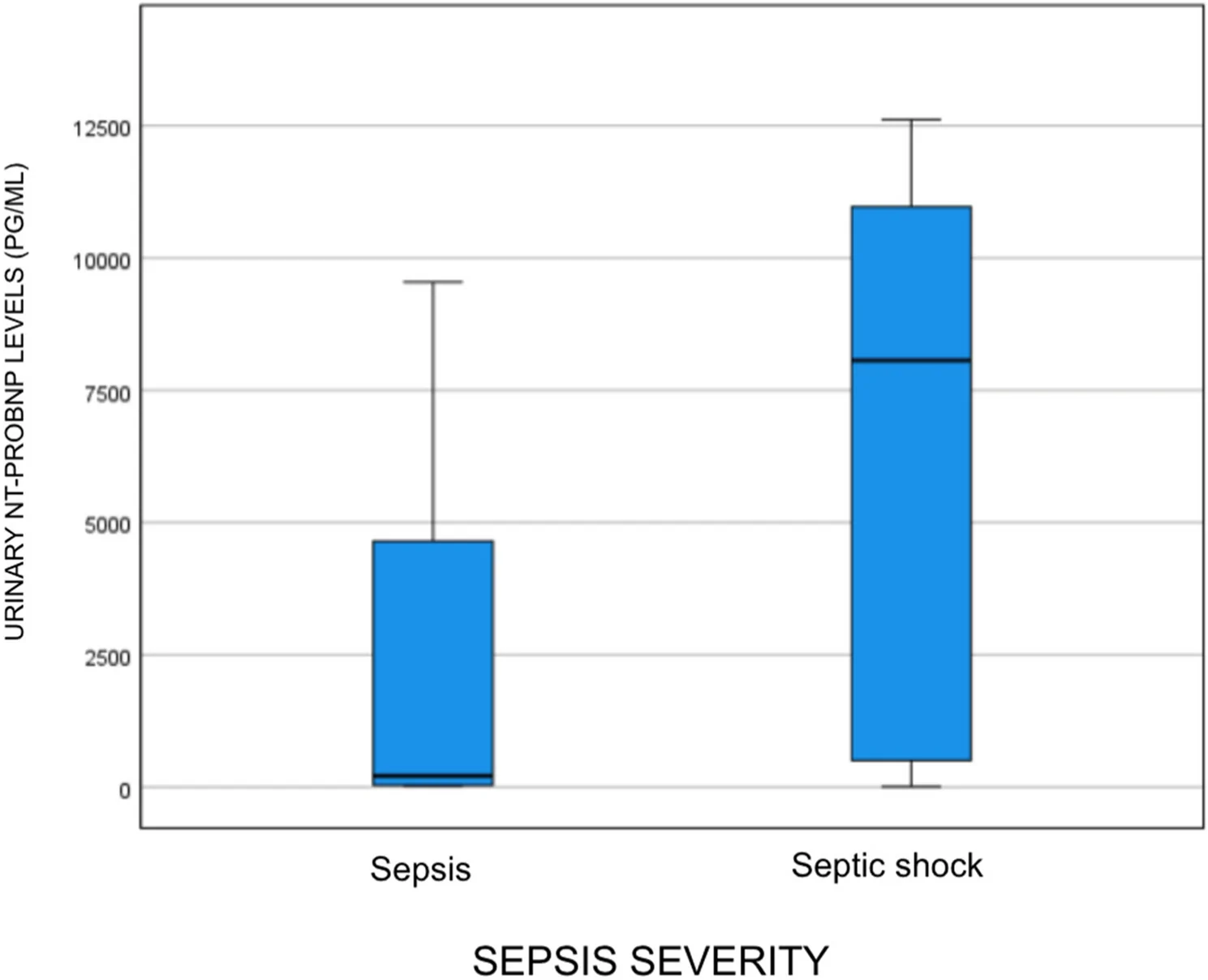
Boxplot of urinary NT-proBNP levels according to sepsis severity in children. Urinary NT-proBNP concentrations are expressed in pg/mL. The central line represents the median, the box indicates the interquartile range (IQR), and the whiskers represent the minimum and maximum values

**Fig. 3:**
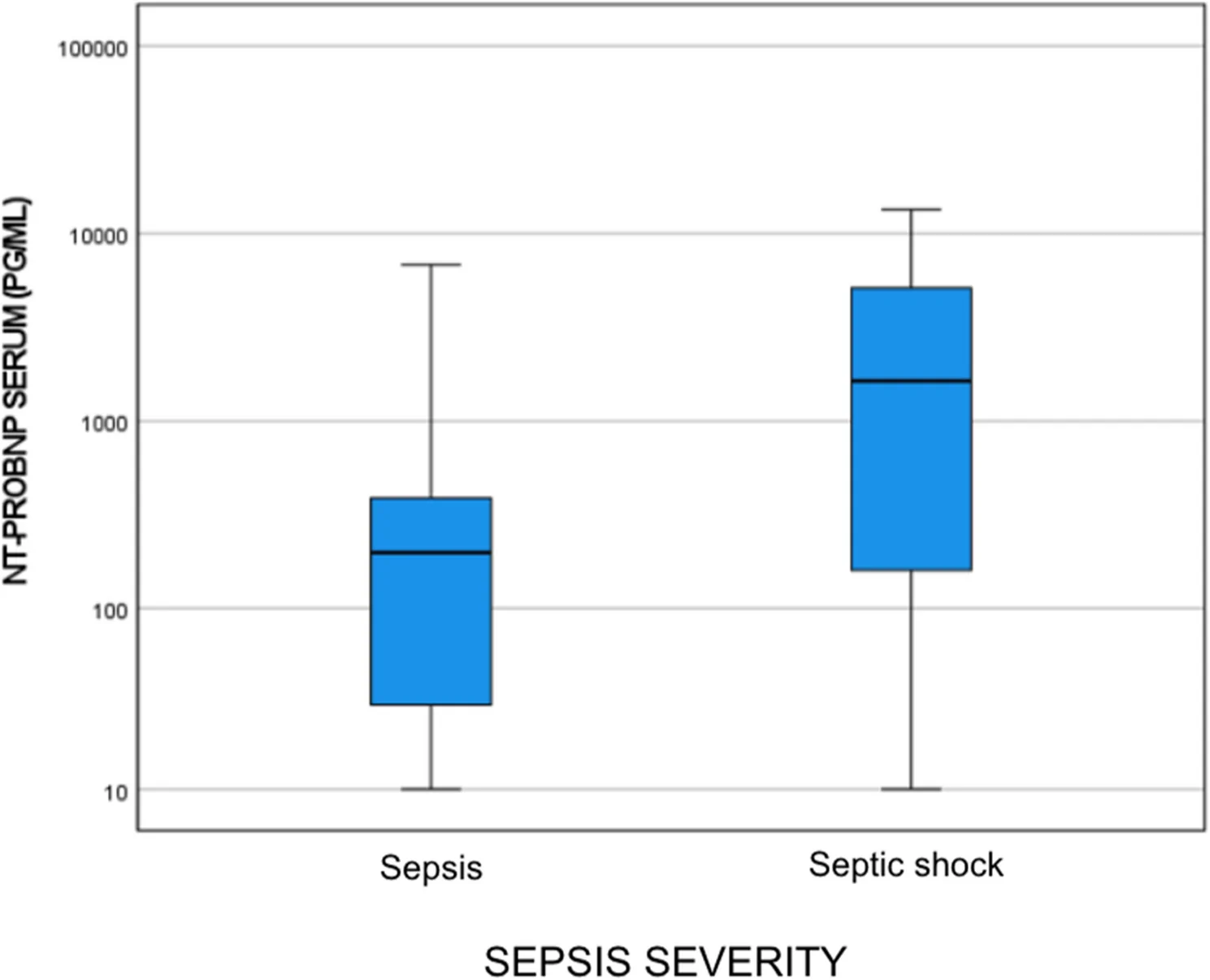
Boxplot of serum NT-proBNP levels according to sepsis severity in children. Serum NT-proBNP concentrations are expressed in pg/mL. The central line represents the median, the box indicates the interquartile range (IQR), and the whiskers represent the minimum and maximum values. The Y-axis is displayed on a logarithmic scale because of the skewed distribution of NT-proBNP values

The results of this research demonstrated that compared to children with sepsis, those with septic shock had much higher amounts of NT-proBNP in their urine and serum. The median urinary NT-proBNP levels in the septic shock group were 8,065.0 pg/mL (IQR 470.0–11,020.0), while in the sepsis group they were 210.0 pg/mL (IQR 37.5–4,710.0) (p = 0.003). Similarly, the median serum NT-proBNP levels in the septic shock group compared to the sepsis group were 1,655.0 pg/mL (IQR 150.0–5,372.5) vs. 200.0 pg/mL (IQR 30.0–397.5) (p = 0.005). All the comparative analyses’ findings are shown in Table 3; Figs. 2 and 3.

**Table 3:** Differences in urinary and serum NT-proBNP levels between sepsis and septic shock patients

Variables	Total (*n* = 52)	Sepsis (*n* = 26)	Septic Shock (*n* = 26)	*P* value
Urinary NT-proBNP level (pg/mL), median (IQR)	915.0 (IQR 77.5–9167.5)	210.0 (IQR 37.5–4,710.0)	8,065.0 (IQR 470.0–11,020.0)	0.003*
Serum NT-proBNP level (pg/mL), median (IQR)	270.0 (IQR 67.5– 3,290.0)	200.0 (IQR 30.0–397.5)	1,655.0 (IQR 150.0–5,372.5)	0.005*

Analysis via the Mann–Whitney test

*significant when *p* ≤ 0.05

### Discriminative performance of urinary and serum NT-proBNP as adjunctive biomarkers for pediatric septic shock classification

**Fig. 4:**
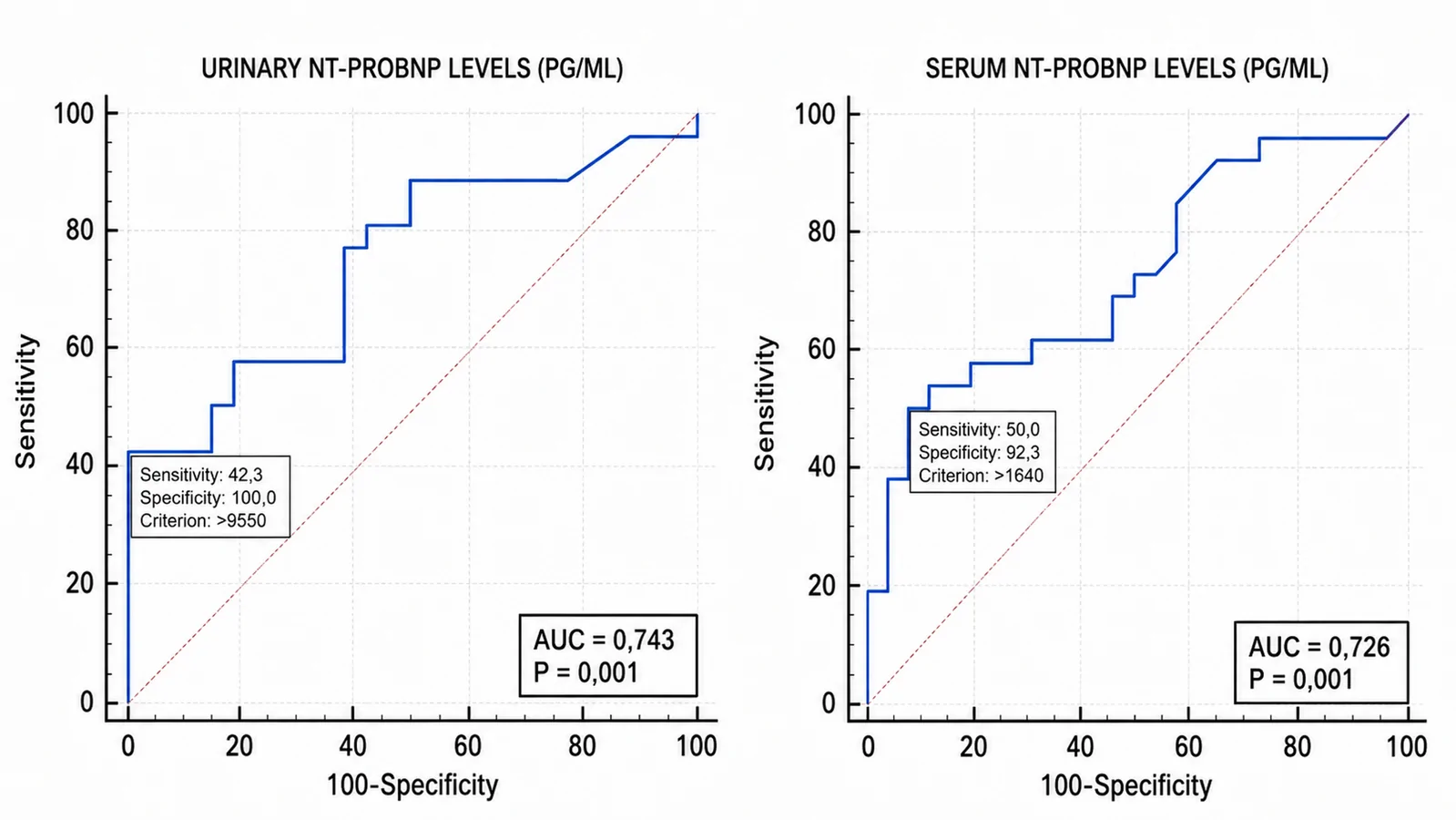
ROC analyses of NT-proBNP levels with septic shock occurrence

To evaluate the diagnostic performance in differentiating septic shock from sepsis, we performed ROC analysis and determined optimal cutoff using the Youden index. Urinary NT-proBNP had an AUC of 0.743 (95% CI 0.603–0.854; *p* = 0.001), whereas serum NT-proBNP had an AUC of 0.726 (95% CI 0.585–0.841; *p* = 0.002). For urinary NT-proBNP, the optimal cut-off value according to the Youden index was > 9,550 pg/mL. There was a 100% PPV and a 63.4% NPV, in addition to a 42.3% sensitivity and a 100% specificity, according to the data. Additionally, when the cut-off value was > 1,640 pg/mL, serum NT-proBNP exhibited a sensitivity of 50.0%, specificity of 92.3%, PPV of 86.7%, and NPV of 64.9%. Its positive likelihood ratio (LR+) was 6.50, whereas the negative likelihood ratio (LR-) was 0.54. The results of the ROC analyses are shown in Table 4; Fig. 4.

**Table 4:** ROC analyses of NT-proBNP levels and septic shock occurrence

	Urinary NT-proBNP	Serum NT-proBNP
	*N* = 52	*N* = 52
AUC (95% CI)	0.743 (0.603–0.854)	0.726 (0.585–0.841)
*P* value	0.001*	0.002*
Cut-off	> 9,550	> 1,640
Sn (%) (95% CI)	42.3 (23.4–63.1)	50.0 (29.9–70.1)
Sp (%) (95% CI)	100.0 (86.8–100.0)	92.3 (74.9–99.1)
PPV (%) (95% CI)	100.0 (∞**)	86.7 (61.9–96.3)
NPV (%) (95% CI)	63.4 (55.5–70.7)	64.9 (55.3–73.4)
LR+ (95% CI)	∞**	6.50 (1.63–25.99)
LR- (95% CI)	0.58 (0.42–0.80)	0.54 (0.36–0.81)

Optimal cut-off values were determined using the Youden index

*AUC *Area Under the Curve, *ROC CI*   95% confidence interval calculated using the standard ROC method, *Sn *Sensitivity, *Sp *Specificity, *PPV *Positive Predictive Value, *NPV *Negative Predictive Value, *LR +*  Positive Likelihood Ratio, *LR*- Negative Likelihood Ratio

*significant when *p* ≤ 0.05

**infinite due to specificity 100%

To assess the stability of the ROC estimates, bootstrap resampling using 1,000 bias-corrected and accelerated (BCa) iterations was performed. Bootstrap AUC values were identical to the original estimates and demonstrated comparable confidence intervals, indicating stable discriminative performance despite the relatively small sample size. Detailed bootstrap methodology and complete results are presented in Additional file 1.

Fragility analysis demonstrated low-to-moderate robustness of the associations between urinary and serum NT-proBNP cut-off values and pediatric septic shock. Detailed fragility analyses, including the 2 × 2 contingency tables, Fragility Index (FI), and Fragility Quotient (FQ), are provided in Additional file 2.

Furthermore, a sensitivity analysis restricted to patients whose samples were collected within 6 h after sepsis diagnosis demonstrated findings consistent with the primary analysis. Urinary NT-proBNP demonstrated an AUC of 0.794 (95% CI 0.611–0.917), whereas serum NT-proBNP demonstrated an AUC of 0.739 (95% CI 0.595–0.853), supporting the robustness of the primary ROC results against variation in sampling time. Because urine and serum specimens were not always obtained simultaneously, these early-sampling analyses were conducted using biomarker-specific subsets, resulting in different sample sizes for urinary and serum NT-proBNP. Detailed sensitivity analysis results are provided in Additional file 3.

### Correlation of Urinary and Serum NT-proBNP with Sepsis Severity as Measured by Phoenix Sepsis Score (PSS)

This study analysed the relationships between NT-proBNP levels and PSS total scores across all subjects. Urinary NT-proBNP levels showed a weak but statistically significant positive correlation with PSS (*r* = 0.261; *p* = 0.031), whereas serum NT-proBNP demonstrated a negligible correlation and did not reach statistical significance (*r* = 0.221; *p* = 0.058).

To evaluate whether this association was driven primarily by cardiovascular dysfunction, additional analyses were performed after excluding the cardiovascular domain from the Phoenix Sepsis Score. Following exclusion of the cardiovascular domain, the previous correlation between urinary NT-proBNP and PSS was no longer observed (*r* = 0.133; *p* = 0.173). Similarly, serum NT-proBNP did not correlate significantly with the modified score (*r* = 0.094; *p* = 0.253).

In the subgroup of patients whose samples were collected within 6 h after sepsis diagnosis, urinary NT-proBNP demonstrated a moderate positive correlation with Phoenix Sepsis Score (*r* = 0.445; *p* = 0.006). However, this association weakened and lost statistical significance after exclusion of the cardiovascular domain (*r* = 0.274; *p* = 0.068). Serum NT-proBNP remained not-significantly correlated with either PSS or the modified score excluding the cardiovascular domain. As urine and serum sampling times were not always concurrent, the urinary and serum ≤ 6-hour subgroup analyses were based on separate biomarker-specific subsets.

**Fig. 5:**
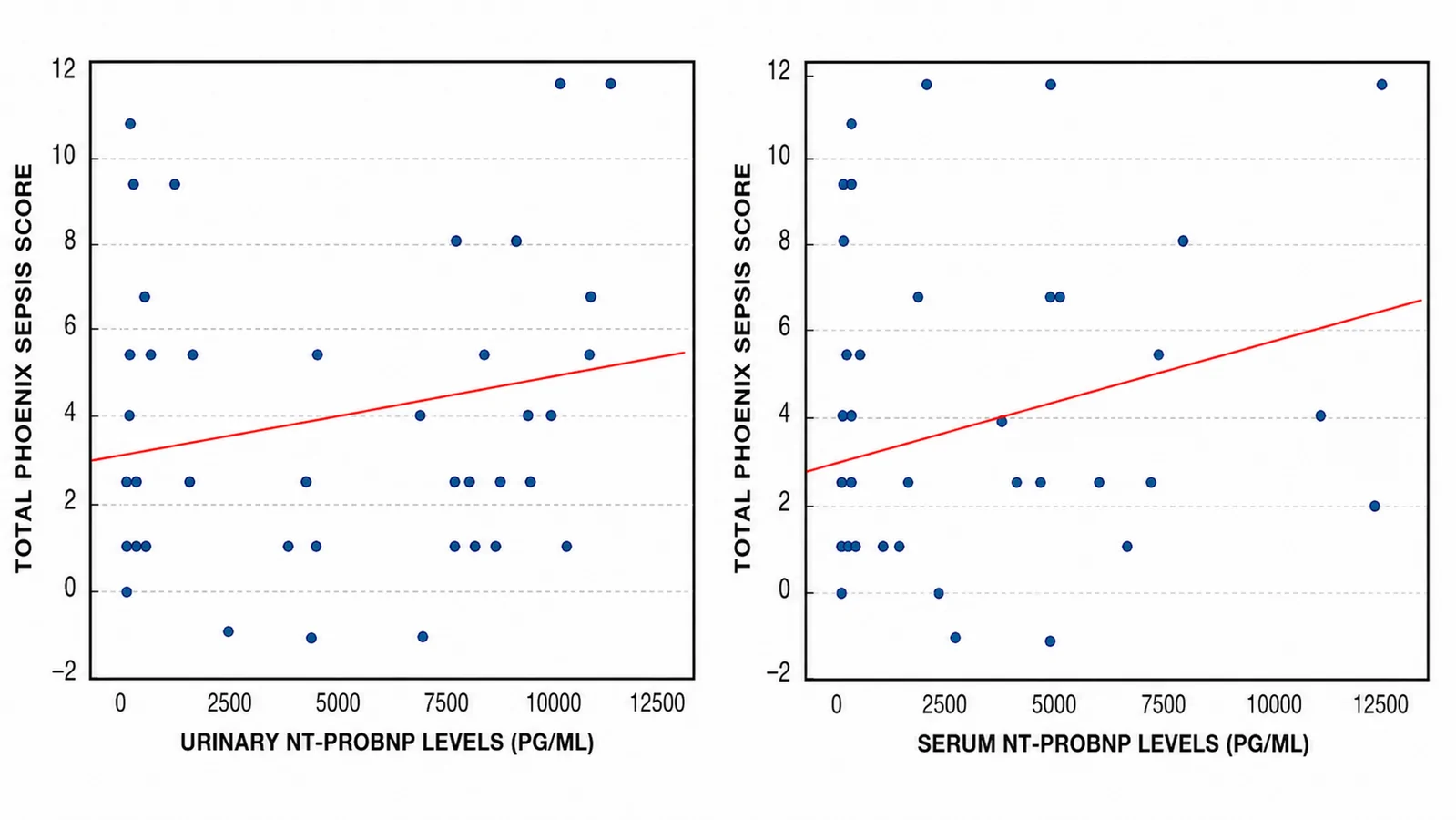
Scatterplot of the correlation between NT-proBNP levels and phoenix sepsis score

These results suggest that the observed association between NT-proBNP, particularly urinary NT-proBNP, and sepsis severity is largely driven by the cardiovascular component of the Phoenix Sepsis Score. The primary correlation analysis is presented in Table 5, whereas additional sensitivity analyses, including correlations after exclusion of the cardiovascular domain and analyses restricted to the ≤ 6-hour sampling subgroup, are provided in Additional file 3. The scatter plots are shown in Fig. 5.

**Table 5:** Correlation between NT-proBNP levels and Phoenix sepsis score

Variable	*n*	Total Phoenix sepsis score		Total Phoenix sepsis score excluding the cardiovascular domain	
		Correlation coefficient (*r*)	*P* value	Correlation coefficient (*r*)	*P* value
Urinary NT-proBNP level (pg/mL)	52	0.261	0.031*	0.133	0.173
Serum NT-proBNP level (pg/mL)	52	0.221	0.058	0.094	0.253

Analysis via *r*ank Spearman’s correlation

*significant when *p*≤0.05

### Sensitivity analysis adjusting for sampling time

Logistic regression analyses adjusted for sampling time demonstrated that both urinary and serum NT-proBNP remained significantly associated with septic shock. After adjustment, log-transformed urinary NT-proBNP showed an adjusted odds ratio (OR) of 2.06 (95% CI 1.15–3.69; *p* = 0.015), whereas serum NT-proBNP showed an adjusted OR of 3.21 (95% CI 1.45–7.11; *p* = 0.004). Sampling time itself was not significantly associated with septic shock. These findings suggest that the observed associations were not substantially explained by variation in sample collection timing. Detailed results of the adjusted logistic regression analyses are provided in Additional file 3.

## Discussion

### Principal findings

This study demonstrated that both urinary and serum NT-proBNP levels were significantly higher in children with septic shock than in those with sepsis. Urinary NT-proBNP showed moderate discriminative performance for differentiating septic shock from sepsis (AUC 0.743), which was comparable to serum NT-proBNP (AUC 0.726). Both biomarkers demonstrated high specificity but limited sensitivity, suggesting bigger potential as adjunctive confirmatory (rule-in) rather than screening biomarkers. In addition, urinary NT-proBNP showed a weak but statistically significant positive correlation with PSS, whereas serum NT-proBNP demonstrated a similar but non-significant trend.

These findings suggest that urinary NT-proBNP may serve as a non-invasive adjunctive biomarker reflecting cardiovascular stress in pediatric sepsis. Although its correlation with overall sepsis severity was weak, its discriminative performance was comparable to that of serum NT-proBNP, supporting its potential utility as a clinically feasible alternative specimen for adjunctive classification of sepsis severity in cross-sectional clinical assessment. Additional robustness analyses supported these findings. Bootstrap resampling yielded nearly identical AUC estimates and confidence intervals, while fragility analysis suggested moderate stability of the associations with septic shock despite the limited sample size.

### Subject characteristics

The study population was predominantly male, which is consistent with previous reports describing a slight male predominance among pediatric sepsis patients [2, 3, 13, 14]. Malnutrition was common in both groups, reflecting the vulnerability of children with severe infections [12]. Pneumonia was the most frequent source of infection, followed by urinary tract infections, in agreement with previous studies identifying respiratory tract infections as the leading cause of pediatric sepsis [2]. However, differences in initial infection diagnoses and respiratory support modalities were not intended as primary comparative endpoints because respiratory support requirements may reflect disease progression and clinical management rather than independent baseline determinants.

Baseline characteristics potentially influencing NT-proBNP concentrations, including age, eGFR, and sampling time, were comparable between the sepsis and septic shock groups. Age-related physiological variation is known to affect NT-proBNP levels, while renal function may influence NT-proBNP clearance [15, 16]. The absence of significant between-group differences in these variables reduces the likelihood that they substantially contributed to the observed differences in NT-proBNP concentrations. Sampling times for serum and urine specimens were also similar between groups, reducing, although not completely eliminating, the possibility of timing-related measurement variability [17, 18]. Previous studies have shown that NT-proBNP levels might rise rapidly in response to cardiovascular stress and correlate with disease severity [9, 17, 19, 20]. Therefore, the absence of significant between-group differences in sampling times reduces, but does not completely eliminate, the possibility that timing-related variability influenced NT-proBNP measurements. Although the primary objective was to evaluate discriminative performance rather than causality, additional sensitivity analyses adjusting for sampling time yielded results consistent with the primary analyses, suggesting that sampling-time variability did not substantially influence the observed associations.

As expected, patients with septic shock showed more severe clinical conditions, including lower MAP, higher PSS total scores, and higher need for invasive mechanical ventilation. The median PSS total scores of 2 in the sepsis group aligns with the baseline threshold required for diagnosing pediatric sepsis, while significantly higher median PSS of 5 in the septic shock group indicates more severe, multi-system organ dysfunction. Furthermore, vasopressor use was observed exclusively in the septic shock group, which aligns with PSS criteria. These findings are consistent with contemporary definitions of septic shock and reflect greater haemodynamic dysfunction and organ failure burden in this group [11, 18, 21–24]. 

### Differences in urinary and serum NT-proBNP levels between sepsis and septic shock in children

Serum NT-proBNP levels are much higher in sepsis patients compared to non-septic individuals, according to several prior investigations [15, 20, 24–26]. Nevertheless, there is a lack of research that compares urinary and serum NT-proBNP levels in children, particularly those with sepsis or septic shock. Children with septic shock demonstrated higher urinary and serum NT-proBNP levels than those with sepsis, supporting an association between NT-proBNP elevation and more significant illness severity [18, 22, 23]. Myocardial stress and circulatory dysfunction are hallmarks of shock, and elevated NT-proBNP levels in septic shock are likely associated with these [18, 19, 22, 27]. In septic shock and more advanced haemodynamic dysfunction, systemic inflammation, proinflammatory cytokine release, persistent hypotension, and impaired tissue perfusion can increase ventricular wall stress and stimulate NT-proBNP secretion [18, 22, 28]. This may explain why higher NT-proBNP levels in the septic shock group indicate more advanced haemodynamic dysfunction than in the sepsis group [22, 27]. 

Although previous studies did not specifically evaluate NT-proBNP according to contemporary Phoenix sepsis and septic shock classifications, they have consistently reported associations between elevated NT-proBNP levels, cardiovascular dysfunction, myocardial impairment, and adverse outcomes in septic patients. These observations provide biological support for the higher NT-proBNP concentrations observed among children with septic shock in the present study [16–20, 22, 23]. These observations provide biological plausibility for the higher urinary and serum NT-proBNP levels observed among children with septic shock in the present study. Although NT-proBNP is commonly used as a biomarker for heart failure, its elevation in sepsis suggests that it may also reflect cardiovascular stress and haemodynamic dysfunction associated with severe systemic inflammation [22, 24, 26]. In line with this, NT-proBNP may have potential utility as an adjunctive biomarker reflecting haemodynamic stress in pediatric sepsis [16, 17, 23]. 

### Discriminative ability of urinary and serum NT-proBNP to differentiate septic shock from sepsis in children

The AUC values of both biomarkers (urinary NT-proBNP AUC 0.743 vs. serum 0.726) were categorized within moderate accuracy or acceptable discrimination, indicating that NT-proBNP levels in both urine and serum demonstrated moderate discriminative performance in assessing pediatric septic shock. The comparable performance of urinary and serum NT-proBNP observed in this study is biologically possible because NT-proBNP is partly cleared by the kidneys and can be detected in urine. Previous studies have reported correlations between urinary and circulating NT-proBNP concentrations, supporting the concept that urinary NT-proBNP may serve as a non-invasive additional indicator of systemic cardiovascular stress [29]. The high specificity and PPV suggest that urinary NT-proBNP may have value as a rule-in adjunctive biomarker. However, the relatively low sensitivity limits its utility as a screening biomarker. The LR- of 0.58 emphasized that the ability of urinary NT-proBNP to rule out septic shock less than the cut-off value is still limited.

The relatively wide confidence intervals surely reflect the limited sample size. Nevertheless, bootstrap resampling demonstrated stable AUC estimates, supporting the internal consistency of the ROC results. Furthermore, fragility analysis indicated that associations with septic shock were low-to-moderately robust, particularly for urinary NT-proBNP, although confirmation in larger cohorts remains necessary.

Moreover, serum NT-proBNP with a cut-off value of > 1,640 pg/mL achieved comparable sensitivity and specificity to urinary measurements. An LR + of 6.50 indicated that values above the cut-off were associated with a higher likelihood of septic shock status. However, the LR- of 0.54, which was comparable with the urinary results, also emphasized that its ability to exclude septic shock at negative results is still not strong enough.

Urinary NT-proBNP demonstrated high specificity but limited sensitivity. Therefore, it may be more appropriately considered an adjunctive confirmatory (rule-in) biomarker rather than a screening tool [30]. On the other hand, the serum NT-proBNP concentration resulted in a relatively comparable performance profile and good LR+ score. However, both biomarkers have limitations in terms of sensitivity and LR- score, so interpretation should be combined with other clinical and laboratory assessments of sepsis severity [17, 18, 22, 23]. 

### Correlation of urinary and serum NT-proBNP with sepsis severity as measured by PSS

A weak but statistically significant correlation was observed between urinary NT-proBNP levels and PSS total scores (*r* = 0.261; *p* = 0.031). However, no statistical significance was found for serum NT-proBNP levels (*r* = 0.221; *p* = 0.058). Higher urinary NT-proBNP concentrations tended to be associated with higher PSS scores. Serum NT-proBNP demonstrated a similar directional trend but did not reach statistical significance. It is biologically likely, as NT-proBNP primarily reflects myocardial stress in sepsis, whereas the PSS represents overall multisystem sepsis severity beyond cardiovascular function [16–18, 28]. Consistent with this, a strong correlation between the two measures is not necessarily expected.

The weak correlation suggests that urinary NT-proBNP reflects a limited component of overall sepsis severity [22, 23]. Moreover, the serum NT-proBNP level also exhibited a similar trend but did not reach statistical significance. These findings suggest that urinary NT-proBNP may reflect certain cardiovascular stress components of pediatric sepsis. However, the weak association with total PSS indicates limited ability to reflect overall multisystem sepsis severity [17, 18, 23, 28]. Scatter plots, as shown in Fig. 5, revealed a positive correlation between NT-proBNP and the PSS, represented by linear regression lines.

Additionally, the association between urinary NT-proBNP and Phoenix Sepsis Score disappeared after exclusion of the cardiovascular domain. This finding suggests that the observed correlation was largely driven by cardiovascular dysfunction rather than overall multisystem sepsis severity. Therefore, NT-proBNP may be better interpreted as a biomarker of cardiovascular stress rather than a global marker of sepsis severity.

### Limitations

This study has several limitations. First, the single-centre design conducted in a tertiary referral hospital and the relatively small sample size may limit the generalizability of the findings to wider pediatric populations and reduce statistical precision. In addition, the cross-sectional design only captured NT-proBNP concentrations during the initial phase of illness, overlooking assessment of longitudinal biomarker trends, prognostic value, treatment response, or causal relationships.

Second, the sampling window was relatively broad, extending up to 48 h after diagnosis for serum samples and 44 h for urine samples. NT-proBNP is a dynamic biomarker that may fluctuate throughout the course of sepsis and septic shock because of evolving haemodynamic stress, disease progression, and therapeutic interventions. Although median sampling times did not differ significantly between groups, variability in collection timing may have contributed to measurement variability. Nevertheless, sensitivity analyses restricted to samples collected within 6 h after sepsis diagnosis yielded findings largely consistent with the primary analyses for septic shock discrimination, suggesting that the main results were reasonably robust to sampling-time variation. Furthermore, urine and serum specimens were not always collected simultaneously in routine clinical practice, resulting in different denominators for the biomarker-specific early-sampling subgroup analyses. In addition, the temporal relationship between biomarker sampling and major therapeutic interventions, including fluid resuscitation, vasopressor initiation, and mechanical ventilation, was not systematically recorded. Consequently, the potential influence of these interventions on NT-proBNP concentrations could not be evaluated. Future studies should consider standardized sampling protocols and adjustment for sampling timing in multivariable analyses.

Third, several NT-proBNP measurements fell outside the assay’s validated quantification range (160–10,000 pg/mL) without repeat testing after appropriate dilution. Measurements beyond the validated range should be interpreted with caution because their analytical accuracy and precision have not been formally established. Furthermore, ELISA measurements were performed according to the manufacturer’s instructions but were not performed in duplicate, which may have introduced additional analytical variability.

Fourth, urinary NT-proBNP concentrations were not normalized to urinary creatinine, urine specific gravity, or other indices of urine concentration. Accordingly, urinary biomarker levels may have been influenced by urine dilution, hydration status, renal handling, and fluid resuscitation, potentially introducing measurement bias. Moreover, echocardiographic assessment was not performed in this study. Consequently, elevated NT-proBNP concentrations could not be directly correlated with objective measures of myocardial dysfunction.

Last, although sensitivity analyses adjusting for sampling time yielded consistent findings, the study was not powered for comprehensive multivariable modelling incorporating additional clinical severity indicators such as invasive mechanical ventilation or vasopressor use. Therefore, residual confounding cannot be excluded. Future studies with larger sample sizes, biomarker normalization strategies, comprehensive cardiac assessment, and multivariable modelling are warranted to further validate our findings.

## Conclusions

NT-proBNP levels in both urine and serum demonstrated moderate ability to support adjunctive cross-sectional discrimination of pediatric septic shock among children with sepsis. Urinary NT-proBNP showed discriminative performance broadly comparable to serum measurements within this study population, with high specificity and modest sensitivity, suggesting that urinary NT-proBNP may have potential value as an adjunctive confirmatory biomarker (rule-in) rather than a screening tool. Furthermore, urinary NT-proBNP may have potential utility as a noninvasive adjunct biomarker alongside serum measurement and clinical parameters, particularly in resource-limited facilities where noninvasive assessment approaches may provide practical advantages. However, given the modest discriminative performance and cross-sectional nature of this study, urinary NT-proBNP should be interpreted as complementary, rather than a replacement for established clinical assessment and severity evaluation tools, and its findings should not be interpreted as evidence of prospective risk stratification or prediction of septic shock development. Multicentre prospective studies with echocardiographic confirmation and serial measurements are needed to validate these findings and to better determine the potential clinical utility of NT-proBNP in pediatric sepsis and septic shock. 

## Supplementary Information

Addiitonal file 1: Additional details relating to bootstrap resampling analysis for ROC curve stability.

Additional file 2: Additional details relating to fragility analysis of significant associations between urinary and serum NT-proBNP and clinical outcomes.

Additional file 3: Additional details relating to supplementary sensitivity analyses evaluating the robustness of NT-proBNP findings.

Additional file 4.

## Data Availability

The raw data supporting the conclusions of this article will be made available by the authors, without undue reservation.

## Abbreviations

NT-proBNP: N-terminal pro B–type natriuretic peptide
PSS: Phoenix sepsis score
IQR: Interquartile range
AKI: Acute kidney injury
CKD: Chronic kidney disease
RRT: Renal replacement therapy
PHCU: Pediatric high care unit
PICU: Pediatric intensive care unit
IMV: Invasive mechanical ventilation
SPSS: Statistical Package for the Social Sciences
BMI: Body mass index
eGFR: Estimated glomerular filtration rate
NYHA: New York Heart Association
ROC: Receiver operating characteristic
AUC: Area under the curve
CI: Confidence interval
PPV: Positive predictive value
NPV: Negative predictive value
LR+: Positive likelihood ratio
LR-: Negative likelihood ratio
MAP: Mean arterial pressure
MDRO: Multidrug-resistant organism
UTI: Urinary tract infection
OR: Odds ratio
BCa: Bias-corrected and accelerated
FI: Fragility index
FQ: Fragility quotient
