# Supplementary material for: Urinary NT-proBNP as a noninvasive biomarker for pediatric septic shock: a comparative study with serum levels
Source: BMC Pediatr. 2026 Jul 29;26:1063. doi: 10.1186/s12887-026-07371-8 (PMC13712029; doi:10.1186/s12887-026-07371-8)
Supplement: Supplementary file 1 [file 12887_2026_7371_MOESM1_ESM.docx]

**Additional file 1. Bootstrap Resampling Analysis for ROC Curve Stability**

**Bootstrap resampling analysis**

To evaluate the stability of the receiver operating characteristic (ROC) estimates obtained from a relatively small sample, bootstrap resampling was performed using the Bias-Corrected and Accelerated (BCa) method with 1,000 iterations. Stability was assessed by comparing the bootstrap-derived area under the curve (AUC) and its 95% BCa confidence interval (CI) with the corresponding estimates from the primary ROC analysis. Comparable AUC values and overlapping confidence intervals were considered indicative of stable discriminative performance.

**Results**

Bootstrap analysis demonstrated excellent agreement with the primary ROC analysis across all evaluated models. For the discrimination of pediatric septic shock, urinary NT-proBNP yielded an identical AUC of 0.743 (95% BCa CI, 0.584–0.867), while serum NT-proBNP yielded an AUC of 0.726 (95% BCa CI, 0.573–0.852). These bootstrap confidence intervals were comparable to those obtained in the primary ROC analysis, supporting the robustness of the estimated discriminative performance.

Overall, the bootstrap resampling analysis demonstrated that repeated resampling did not materially alter the ROC estimates, supporting the stability and reliability of the discriminative performance despite the relatively limited sample size.

**Table S1.** **Bootstrap Resampling Results of ROC Analyses for NT-proBNP in Septic Shock**

| Analysis | Original AUC  (95% CI) | Bootstrap AUC (95% BCa CI) | Interpretation |
| --- | --- | --- | --- |
| Urinary NT-proBNP for septic shock | 0.743 (0.603–0.854) | 0.743 (0.584–0.867) | Very stable |
| Plasma/Serum NT-proBNP for septic shock | 0.726 (0.585–0.841) | 0.726 (0.573–0.852) | Very stable |

Abbreviations: AUC = *area under the curve*; BCa = *bias-corrected and accelerated*; BCa CI = *95% bias-corrected and accelerated bootstrap confidence interval (1,000 bootstrap iterations).*
