# Supplementary material for: Urinary NT-proBNP as a noninvasive biomarker for pediatric septic shock: a comparative study with serum levels
Source: BMC Pediatr. 2026 Jul 29;26:1063. doi: 10.1186/s12887-026-07371-8 (PMC13712029; doi:10.1186/s12887-026-07371-8)
Supplement: Supplementary file 2 [file 12887_2026_7371_MOESM2_ESM.docx]

**Additional file 2. Fragility Analysis of the Associations Between Urinary and Serum NT-proBNP and Septic Shock**

**Fragility analysis**

To evaluate the robustness of statistically significant dichotomous associations obtained from the primary analyses, fragility analysis was performed using the Fragility Index (FI) and Fragility Quotient (FQ). The FI represents the minimum number of outcome reassignments required to change a statistically significant result (*p* < 0.05) into a non-significant result (*p* ≥ 0.05). The FQ was calculated by dividing the FI by the total study population (N = 52). Larger FI and FQ values indicate greater robustness of the findings, whereas smaller values suggest that statistical significance is more susceptible to small changes in the data.

**Results**

**Association with pediatric septic shock**

**Table S21. The 2×2 table analysis and fragility index based on NT-proBNP cut-off for septic shock**

| Variable | Septic shock | Sepsis | Fisher *p* | FI | FQ | Interpretation |
| --- | --- | --- | --- | --- | --- | --- |
| Serum NT-proBNP levels (pg/mL) |  |  | 0.002 | 3 | 0.058 | Low-to-moderate robustness |
| >1,640 | 13 | 2 |  |  |  |  |
| ≤1,640 | 13 | 24 |  |  |  |  |
| Urinary NT-proBNP levels (pg/mL) |  |  | <0.001 | 4 | 0.077 | Moderate robustness |
| >9,550 | 11 | 0 |  |  |  |  |
| ≤9,550 | 15 | 26 |  |  |  |  |

Note: FI = Fragility Index; FQ = Fragility Quotient. FI represents the minimum number of outcome status changes required to shift a statistically significant result to non-significance (p≥0.05). FQ is calculated using the formula FI/N, where N=52. The higher the FI and FQ values, the more robust the analysis result; conversely, small FI and FQ values indicate results that are more susceptible to minor changes in the data. Generally, an FI of 0–1 indicates highly fragile results, 2–4 indicates low-to-moderate robustness, while an FI ≥5 indicates relatively more robust results. An FQ <0.05 indicates highly fragile results, 0.05–0.10 indicates moderate robustness, and an FQ >0.10 indicates relatively more stable results.

Fragility analysis demonstrated low-to-moderate robustness for the associations between NT-proBNP concentrations and pediatric septic shock. Using the ROC-derived cut-off value of >1,640 pg/mL, serum NT-proBNP was significantly associated with septic shock (Fisher's exact *p* = 0.002), with an FI of 3 and an FQ of 0.058. This indicates that reassignment of the outcome in three participants (5.8% of the study population) would be required to eliminate statistical significance.

Similarly, urinary NT-proBNP using a cut-off value of >9,550 pg/mL was significantly associated with septic shock (Fisher's exact *p* < 0.001), with an FI of 4 and an FQ of 0.077. All participants with urinary NT-proBNP concentrations above this cut-off were classified as having septic shock, whereas no patients in the sepsis group exceeded the cut-off value. Thus, statistical significance would change only after reassignment of four participants (7.7% of the study population), indicating slightly greater robustness than that observed for serum NT-proBNP.

Overall, these findings suggest that the associations between urinary and serum NT-proBNP and pediatric septic shock remained relatively stable despite the limited sample size, although confirmation in larger cohorts remains warranted.
