# Supplementary material for: Urinary NT-proBNP as a noninvasive biomarker for pediatric septic shock: a comparative study with serum levels
Source: BMC Pediatr. 2026 Jul 29;26:1063. doi: 10.1186/s12887-026-07371-8 (PMC13712029; doi:10.1186/s12887-026-07371-8)
Supplement: Supplementary file 3 [file 12887_2026_7371_MOESM3_ESM.docx]

**Additional file 3. Sensitivity Analyses Assessing the Robustness of NT-proBNP Findings**

**Description**

This supplementary file presents additional sensitivity analyses evaluating the robustness of the primary findings. These include (1) receiver operating characteristic (ROC) analyses restricted to samples collected within 6 hours after sepsis diagnosis, (2) logistic regression analyses adjusted for sampling time, and (3) correlation analyses after exclusion of the cardiovascular domain from the Phoenix Sepsis Score.

**1. ROC Analysis Restricted to Samples Collected Within 6 Hours After Sepsis Diagnosis**

**Table 7. ROC Analysis of Initial Urinary and Serum NT-proBNP Levels for Pediatric Septic Shock in the ≤6-Hour Sampling Subgroup**

|  | **Urinary NT-proBNP** | **Serum NT-proBNP** |
| --- | --- | --- |
|  | **N=31** | **N=50** |
| **AUC (95% CI)** | 0.794 (0.611–0.917) | 0.739 (0.595–0.853) |
| ***P* value** | <0.001* | 0.001* |
| **Cut-off** | >4,890 | >1,640 |
| **Sn (%) (95% CI)** | 64.7 (38.3–85.8) | 50.0 (29.9–70.1) |
| **Sp (%) (95% CI)** | 92.9 (66.1–99.8) | 95.8 (78.9–99.9) |
| **PPV (%) (95% CI)** | 91.7 (61.7–98.7) | 92.9 (64.8–98.9) |
| **NPV (%) (95% CI)** | 68.4 (52.8–80.7) | 63.9 (54.4–72.4) |
| **LR+ (95% CI)** | 9.06 (1.33–61.86) | 12.00 (1.70–84.92) |
| **LR- (95% CI)** | 0.38 (0.20–0.74) | 0.52 (0.35–0.77) |

AUC=*Area Under Curve*, CI=*Confidence Interval,* Sn=*Sensitivity*, Sp=*Specificity*, PPV=*Positive Predictive Value*, NPV=*Negative Predictive Value*, LR=*Likelihood Ratio.* *significant when *p* ≤ 0.05

**Interpretation**

Restriction of the analysis to samples collected within 6 hours after sepsis diagnosis produced findings largely consistent with the primary analysis. Urinary NT-proBNP demonstrated an AUC of 0.794 (95% CI 0.611–0.917), whereas serum NT-proBNP demonstrated an AUC of 0.739 (95% CI 0.595–0.853), supporting the robustness of the primary findings with respect to sampling-time variation.

**2. Logistic Regression Adjusted for Sampling Time**

**Table 10. Logistic Regression Analysis of Urinary and Serum NT-proBNP for Pediatric Septic Shock After Adjustment for Sampling Time (≤ 6-hour sample subgroup)**

| **Outcome** | **Model** | **Variabel** | **Adjusted OR** | **95% CI** | ***P* value** |
| --- | --- | --- | --- | --- | --- |
| Septic Shock | Serum NT-proBNP | Log NT-proBNP (serum) | 3.212 | 1.451–7.111 | 0.004* |
|  |  | Serum sampling time | 0.653 | 0.336–1.271 | 0.210 |
|  | Urinary NT-proBNP | Log NT-proBNP (urine) | 2.063 | 1.153–3.691 | 0.015* |
|  |  | Urine sampling time | 0.996 | 0.944–1.051 | 0.892 |

**Note:** Binary logistic regression analysis was performed using the Enter method. NT-proBNP values were log10-transformed before analysis. Adjusted odds ratios (ORs) were calculated after controlling for sample collection timing. *P* $\leq$ 0.05 was considered statistically significant.

**Interpretation**

After adjustment for sampling time, both urinary and serum NT-proBNP remained independently associated with pediatric septic shock. Log-transformed serum NT-proBNP showed an adjusted odds ratio (OR) of 3.212 (95% CI 1.451–7.111; *p*=0.004), whereas urinary NT-proBNP demonstrated an adjusted OR of 2.063 (95% CI 1.153–3.691; *p*=0.015). Sampling time itself was not significantly associated with septic shock in either model.

These findings indicate that the observed associations between NT-proBNP concentrations and pediatric septic shock were not substantially explained by variation in sample collection timing.

**3. Correlation Analysis After Exclusion of the Cardiovascular Domain from the Phoenix Sepsis Score**

**Table 9. Correlation Between Urinary and Serum NT-proBNP Levels and the Phoenix Sepsis Score in the ≤6-Hour Sampling Subgroup**

| **Variable** | **n** | **Total Phoenix sepsis score** | | **Phoenix sepsis score excluding the cardiovascular domain** | |
| --- | --- | --- | --- | --- | --- |
|  |  | **Correlation coefficient (r)** | ***P* value** | **Correlation coefficient (r)** | ***P* value** |
| **Urinary NT-proBNP level (pg/mL)** | 31 | 0.445 | 0.006* | 0.274 | 0.068 |
| **Serum NT-proBNP level (pg/mL)** | 50 | 0.234 | 0.051 | 0.096 | 0.254 |

Notes: Analysis via *rank Spearman’s correlation*, *significant when *p* $\leq$ 0.05

**Interpretation**

Across the entire cohort, urinary NT-proBNP demonstrated a weak but statistically significant correlation with the total Phoenix Sepsis Score, whereas serum NT-proBNP did not. After exclusion of the cardiovascular domain, neither biomarker remained significantly correlated with the modified score.

Among patients whose samples were collected within 6 hours after diagnosis, urinary NT-proBNP remained moderately correlated with the total Phoenix Sepsis Score (*r*=0.445, *p*=0.006). However, this association weakened and lost statistical significance after exclusion of the cardiovascular domain (*r*=0.274, *p*=0.068). Serum NT-proBNP remained non-significantly correlated with both the original and modified Phoenix Sepsis Scores.

These findings suggest that the observed relationship between NT-proBNP, particularly urinary NT-proBNP, and sepsis severity is largely driven by the cardiovascular component of the Phoenix Sepsis Score.
