# Supplementary material for: Urinary NT-proBNP as a noninvasive biomarker for pediatric septic shock: a comparative study with serum levels
Source: BMC Pediatr. 2026 Jul 29;26:1063. doi: 10.1186/s12887-026-07371-8 (PMC13712029; doi:10.1186/s12887-026-07371-8)
Supplement: Supplementary file 4 [file 12887_2026_7371_MOESM4_ESM.docx]

STROBE Statement—Checklist of items that should be included in reports of cross-sectional studies
Items
No
Recommendation
Check-list
Notes
Title and abstract
1
(a) Indicate the study’s design with a commonly used term in the title or the abstract
√
The study design is stated as a prospective cross-sectional analytical study in the Methods section of the abstract.
(b) Provide in the abstract an informative and balanced summary of what was done and what was found
The abstract provides a clear summary including background rationale, study design, eligibility criteria, measurement methods, statistical analyses, key findings (AUC with 95% CI, sensitivity, specificity), and cautious conclusions. 
Introduction


Background/rationale
2
Explain the scientific background and rationale for the investigation being reported
√
Scientific background includes the burden of pediatric sepsis, lack of Phoenix Sepsis Score’s (PSS) sensitivity in detecting early cardiovascular risk, the mechanism behind NT-proBNP release under myocardial strain, and its potential of urinary measurement as an additional noninvasive biomarker for pediatric sepsis severity. The research gap regarding these matters is clearly stated.
Objectives
3
State specific objectives, including any prespecified hypotheses
√
Three main objectives comprise urinary and serum NT-proBNP level comparison between pediatric sepsis and septic shock patients, their discriminative ability using ROC analysis, and their correlations with PSS score.  
Methods


Study design
4
Present key elements of study design early in the paper
√
It is stated as a prospective cross-sectional analytical study conducted at a tertiary referral centre.
Setting
5
Describe the setting, locations, and relevant dates, including periods of recruitment, exposure, follow-up, and data collection
√
The study was conducted at Dr. Hasan Sadikin General Hospital, Bandung, Indonesia from December 2025 to January 2026. Patients were consecutively from various points of care at the hospital. Samples were collected within 48 hours of sepsis diagnosis based on PSS criteria.
Participants
6
(a) Give the eligibility criteria, and the sources and methods of selection of participants
√
Inclusion criteria consisted of children aged 1 month–18 years with sepsis or septic shock based on PSS, NT-proBNP measurements within 48 hours of diagnosis, and obtainment of parental consent. Exclusion criteria comprised known cardiac, renal, or urological abnormalities, and unfit laboratory samples. The selection method was consecutive sampling.
Variables
7
Clearly define all outcomes, exposures, predictors, potential confounders, and effect modifiers. Give diagnostic criteria, if applicable
√
Main exposure variables were both urinary and serum NT-proBNP levels (pg/mL). Primary outcome was septic shock status based on PSS criteria. Secondary outcome was PSS as continuous severity numbers. Potential confounders included age, eGFR, and sampling time for both urine and serum. Diagnostic criteria were based on PSS: sepsis is defined by having suspected or confirmed infection and PSS ≥ 2 points, while septic shock is sepsis that includes ≥ 1 cardiovascular dysfunction point.
Data sources/ measurement
8*
 For each variable of interest, give sources of data and details of methods of assessment (measurement). Describe comparability of assessment methods if there is more than one group
√
Measurement of both urinary and serum NT-proBNP was performed using ELISA-based Elabscience® Human NT-proBNP kit. PSS was used for the sepsis criteria, while relevant baseline characteristics and clinical data were simultaneously obtained. Sepsis and septic shock groups based on PSS criteria were compared for the NT-proBNP differences, ROC analysis, and were correlated to the total PSS points as well.
Bias
9
Describe any efforts to address potential sources of bias
√
Consecutive sampling method was chosen to reduce selection bias. Baseline characteristics were compared between groups for potential confounding factors. Although both groups were similar, we could not guarantee some unmeasured factors did not influence the results. Furthermore, known factors that could affect the measurement level were also excluded from the research. 
Study size
10
Explain how the study size was arrived at
√
For comparison analysis, sample size was calculated using assumed moderate effect size of 0.7 as there was no similar research. One-sided α of 0.05 and a statistical power of 80% yielded the requirement of 26 subjects per group. For correlation analysis, assumed correlation effect size of 0.4 was used and resulted in minimum of 47 subjects overall. Since a total of 52 subjects were required for comparison, this number also meet the minimum requirement of for correlation analysis. 
Quantitative variables
11
Explain how quantitative variables were handled in the analyses. If applicable, describe which groupings were chosen and why
√
NT-proBNP levels were considered as continuous variables. Due to skewed distribution, we reported medians and IQR. PSS total score was also analysed as continuous variables. 
Statistical methods
12
(a) Describe all statistical methods, including those used to control for confounding
√
Shapiro–Wilk test was used for normality test. Mann–Whitney U test was used for group comparison and Spearman’s test was used for the correlation. ROC analysis was used for the discriminative ability with reported AUC and 95% CI, sensitivity, specificity, PPV, NPV, LR+, and LR-. As the primary objective was to evaluate discriminative performance rather than to conclude causality, multivariable analysis was not performed. However, potentially confounding baseline characteristics like age, eGFR, and sampling time variables were comparable between groups.
(b) Describe any methods used to examine subgroups and interactions
No subgroup or interaction analyses beyond sepsis classification, total PSS, and NT-proBNP levels.
(c) Explain how missing data were addressed
No missing data were reported.
(d) If applicable, describe analytical methods taking account of sampling strategy
Consecutive sampling was used in this study, explained in the data collection.
(e) Describe any sensitivity analyses
No additional sensitivity analyses were performed
Results


Participants
13*
(a) Report numbers of individuals at each stage of study—eg numbers potentially eligible, examined for eligibility, confirmed eligible, included in the study, completing follow-up, and analysed
√
There were 59 potentially eligible for our study, but 5 could not meet the inclusion criterion of having the samples taken within 48 hours of diagnosis. Therefore, 54 children met inclusion criteria, and then 1 was excluded because of having previous heart failure NYHA class III and pulmonary hypertension, 1 was excluded because of having previous AKI stage III (failure). Final analyses included 52 children (26 sepsis, 26 septic shock). Since it was a cross-sectional study, there was no loss to follow-up. 
(b) Give reasons for non-participation at each stage
Reasons for exclusion at each stage (not meeting the 48-hour sampling criterion, predefined exclusion criteria) are stated in the Results and flow diagram.
(c) Consider use of a flow diagram
Flow diagram is provided in figure 1.
Descriptive data
14*
(a) Give characteristics of study participants (eg demographic, clinical, social) and information on exposures and potential confounders
√
Baseline characteristics including potential confounders were mentioned and presented in tables 1 and 2
(b) Indicate number of participants with missing data for each variable of interest
There were no missing NT-proBNP values.
Outcome data
15*
Report numbers of outcome events or summary measures
√
Septic shock status (n = 26) was the primary outcome. NT-proBNP levels were reported as medians and IQR, while PSS numbers were also stated.
Main results
16
(a) Give unadjusted estimates and, if applicable, confounder-adjusted estimates and their precision (eg, 95% confidence interval). Make clear which confounders were adjusted for and why they were included
√
Unadjusted estimates including AUC values with 95% CI, sensitivity, specificity, PPV, NPV, and likelihood ratios were reported. No confounder-adjusted estimates were calculated.
(b) Report category boundaries when continuous variables were categorized
Continuous variables were not categorized except where clinically defined (e.g., septic shock classification by PSS). ROC-derived cut-offs were also stated.
(c) If relevant, consider translating estimates of relative risk into absolute risk for a meaningful time period
No translation to absolute risk since it was a cross-sectional study.
Other analyses
17
Report other analyses done—eg analyses of subgroups and interactions, and sensitivity analyses
√
ROC and correlation analyses were performed as secondary. Additional analyses included sensitivity analyses restricted to samples collected within 6 hours after diagnosis, logistic regression adjusted for sampling time, bootstrap resampling of ROC estimates, fragility analyses, and correlation analyses excluding the cardiovascular domain.
Discussion


Key results
18
Summarise key results with reference to study objectives
√
Urinary and serum NT-proBNP levels were significantly higher in septic shock than sepsis. Both biomarkers showed moderate discriminative ability. Urinary NT-proBNP had high specificity and may serve as a confirmatory (rule-in) biomarker. Urinary NT-proBNP also correlated weakly but significantly with PSS, while serum did not.
Limitations
19
Discuss limitations of the study, taking into account sources of potential bias or imprecision. Discuss both direction and magnitude of any potential bias
√
The limitations section discusses potential sources of bias and imprecision, including the single-centre setting, relatively small sample size, cross-sectional design, variability in sampling timing, laboratory-related limitations (including measurements outside the validated assay range without repeat dilution and lack of duplicate ELISA measurements), absence of urinary biomarker normalization and echocardiographic assessment, and the possibility of residual confounding.
Interpretation
20
Give a cautious overall interpretation of results considering objectives, limitations, multiplicity of analyses, results from similar studies, and other relevant evidence
√
Results were interpreted cautiously, positioning NT-proBNP as an adjunctive biomarker rather than a sole diagnostic tool. Findings were discussed in relation to existing research both in children and adults.
Generalisability
21
Discuss the generalisability (external validity) of the study results
√
Findings are applicable to tertiary-care pediatric populations in similar low- to middle-income settings. External validation in multicentre cohorts is needed.
Other information


Funding
22
Give the source of funding and the role of the funders for the present study and, if applicable, for the original study on which the present article is based
√
The study was funded by all the authors. The funders had no role in study design, data collection, analyses, interpretation, or manuscript preparation.

*Give information separately for exposed and unexposed groups.

Note: An Explanation and Elaboration article discusses each checklist item and gives methodological background and published examples of transparent reporting. The STROBE checklist is best used in conjunction with this article (freely available on the Web sites of PLoS Medicine at http://www.plosmedicine.org/, Annals of Internal Medicine at http://www.annals.org/, and Epidemiology at http://www.epidem.com/). Information on the STROBE Initiative is available at http://www.strobe-statement.org
